# Extracting the Relationship and Evolutionary Rule Connecting Residents’ Travel Demand and Traffic Supply Using Multisource Data

**DOI:** 10.3390/s21062179

**Published:** 2021-03-20

**Authors:** Zijia Wang, Zhixiang Chen, Youyin Shi, Liping Huang

**Affiliations:** 1 Department of Civil Engineering, Beijing Jiaotong University, Beijing 100044, China; zjwang@bjtu.edu.cn (Z.W.); 20121169@bjtu.edu.cn (Z.C.); 2 Engineering and Technology Research Center of Rail Transit Line Safety and Disaster Prevention, No. 3 Shangyuancun, Haidian District, Beijing 100044, China; 3 Department of Civil Engineering, Central South University, Changsha 410000, China; youyinshi@csu.edu.cn; 4 School of Electrical and Electronic Engineering, Nanyang Technological University, 50 Nanyang Avenue, Singapore 639798, Singapore

**Keywords:** urban rail transit, fractal approaches, improved traffic assignment model, spatiotemporal evolution, travel demand and traffic supply

## Abstract

Urban rail transit (URT) systems are often regarded as the backbone of their respective city. The evolutionary features of URT systems have attracted much attention in recent years, but their evolution and their distinct function in contrast to other transit modes have seldom been investigated, especially quantitatively from the perspective of work–residence separation. Accordingly, we propose a framework for exploring the evolution of URT topological networks and demand-weighted networks, comparing the different impacts of all transit modes on work–residence separation. In this study, a URT passenger flow assignment model was formulated on the basis of travel cost function and an improved logit model was proposed that takes into account the heterogeneity of passengers. This model was used to generate a section load, which is regarded as a weight and able to reflect the residents’ demand for travel by URT. Then, the fractal dimensions for a non-weighted network and demand-weighted network are proposed and their indications for transportation explained. Finally, the Beijing Subway System (BSS) is used as a case study by employing fifty years of network data and ten years of smart card data. Using fractal approaches, the different characteristics illustrated by the two networks were investigated and the reasons behind the observed patterns explained. In addition, the spatial features of the rail network, in terms of fractal indictors, were compared with population distribution and urban mobility for all modes, extracted from phone data as a proxy. Thus, the relationship between the residents’ travel demand and traffic supply can be revealed to some extent. The main finding of this work is that demand must be taken into account when analyzing the fractal features of a transport network, lest the demand side be separated from the supply and important issues missed such as inconsistencies between demand and supply. Additionally, the role of rail transit in work–home imbalance can be investigated in the context of urban mobility for an entire city.

## 1. Introduction

Public transportation plays an important and indeed indispensable role in daily commuting in almost all cities [1,2,3]. Due to the saturation of ground transport, urban rail transit (URT) systems are preferred by many metropolises to reduce traffic congestion because of their high speed, large capacity, energy efficiency, and use of urban non-surface space [4,5,6]. Moreover, URT systems can promote the development of a city and even reshape urban structure such as by enhancing or mitigating work–residence separation. The topological network of URT systems and their coverage can be seen as the transportation infrastructure supply from an urban planner’s point of view, with the network traffic flow of URT systems representing the demand side [7,8,9]. Increasing traffic demand drives the extension of the URT system, with the new rail lines generating still further demand. The different evolutionary features of topological networks and demand-weighted networks (topological networks loaded by station and section demand) can reflect the supply–demand matching level and the different stages of city growth. However, on a complex rail network, section load cannot be easily retrieved because many Origin-Destination (OD) pairs have more than one reasonable path, and different passengers may prefer different paths. To obtain a relatively accurate section demand between two adjacent stations, a traffic assignment model, often some type of multinominal logit model (MLM), is consistently adopted in the literature, however, passenger heterogeneity is seldom considered. Furthermore, the parameters used in the path choice model are normally calibrated against stated preferences derived from questionnaire data, which might not conform well to real life. Thus, we used Bayesian inference as a more reliable alternative to the traditional questionnaire survey method, with an Markov chain Monte Carlo (MCMC) algorithm used to improve calculation efficiency. In terms of estimated sectional passenger flow, URT development level has been well analyzed using fractal approaches.

There are several methods for evaluating the evolutionary features of a URT. As the fractal approach is an effective way of describing fragmented anthropogenic objects and obtaining scale-independent results [10,11], we used it to assess the evolutionary features of a non-weighted URT network and a transit-weighted network. Fractal concepts were proposed by Mandelbrot [10], and several definitions of fractal dimensions have been introduced to describe fractality in a quantified way, with application to an urban transport network following nearly a decade later. Benguigui [12] first identified the fractal features of an urban transport network and carried out case studies on the London Underground, Paris Metro, Moscow Metro, and Rhine Subway (Germany). Batty [13,14] linked transport analysis to urban geography on the basis of fractal dimensions, after which fractal approaches have been widely applied to different parts of cities including the city center [15], the whole scope of a city [16], and conurbations [11]. Different modes of transport have also been analyzed using fractal methods including traditional surface transport [15], subway [16], and the whole public transport network. Several urban issues are discussed in the research in relation to transport networks such as the complexity and space filling capacity of urban transport networks [17], city morphology [18], and the consistency of transportation and the built-up environment [19]. As this study goes beyond these considerations, with fractal characteristics unable to be properly described using only one fractal dimension, multifractals [19,20,21] were seen as an extension for complicated fractal structures. The existing literature is devoted chiefly to the structure of a transport network without demand on it, with no research addressing the fractal features of a transit network for its entire life from both a topological network perspective and a demand-weighted network perspective. In addition, fractal comparison has rarely been made of an all-mode transit network with respect to work–residence separation within a city. This study focused on the different evolutionary fractal features of a rail topological network and a rail demand-weighted network. Various conclusions have been suggested relating to the dynamic interactions between rail network development and urban expansion. Moreover, the fractal dimensions of URT mode and all modes in Beijing have been calculated to further explore the work–residence separation problem, which has also seldom been addressed in the literature.

Then, a precise and reasonable traffic assignment model is used as the basis for comprehensive analysis of fractal features. Traffic assignment is first introduced to the urban surface network [22]. However, as URT use has increased, traffic assignment of subway systems has drawn increasing attention. This study discusses improvements to passengers’ generalized travel cost function, probability determination for path choices, and calculation efficiency. To capture the passengers’ more complicated path choice behavior in the real world, various stochastic factors and uncertainties have been introduced such as passengers’ preferences [23], crowding or service level [24], and transport network familiarity. In recent years, research concentrating on the generalized cost function has turned to passengers’ heterogeneity, with several assignment models proposed based on passenger clustering [25,26]. However, the socioeconomic attributes of passengers are hard to access, constraining further investigation of the stochastic assignment method. Fortunately, thanks to the widespread use of automatic fare collection (AFC) systems in URT systems, abundant travel information recorded in smart card data (SCD) can be collected. Furthermore, passengers’ spatial and temporal attributes can be derived by mining SCD. Accordingly, an improved traffic assignment model can be devised. Thus, topic model was chosen to distinguish the passengers’ heterogeneity, with *K*-means used to acquire passenger clustering for calculation of the path-chosen probability. As dynamic traffic assignment for the URT system requires high levels of accuracy and efficiency, an effective machine mining method, Bayesian inference [27,28], was adopted to calibrate the parameters of the utility function. Moreover, the passenger attributes already discussed could be used as the input data to differentiate parameter calibration for each passenger clustering. When assessing data of large scale and computational complexity, the MCMC algorithm is selected for its efficiency and accuracy [29].

A public transportation system, especially in big cities, always contains multiple modes. Thus, a single trip could comprise several modes of transport trip–leg. In this study, we further investigated the ways in which URT affects city structure and the different roles that URT systems play in thee spatial imbalance of workplaces and residences, compared with the overall mobility of a whole city. Distinct characteristics of different modes determine the attraction of different passengers to each mode and also reveal or even affect the city’s urban structure in different ways. However, because quantitative measures are rarely devoted to investigating this issue, it should be enlightening to compare the travel features of rail riders using the fractal dimensions of a demand-weighted network, in contrast to that of urban mobility as a whole. To this end, mobile phone base station data were used as a proxy for population distribution and whole-city mobility, serving as a benchmark against which to reveal the distinct features of the rail transit network and its effects on urban development. The main contributions of this study are as follows: (i) An improved traffic assignment model that takes into account the spatiotemporal characteristics of passengers is introduced. (ii) A fifty-year time span fractal analysis of a rail topological network and a ten-year time span fractal analysis of a demand-weighted rail network are carried out, revealing long-term evaluation patterns of a rail network with or without passenger load. (iii) A comprehensive investigation of fractal features for rail transit in Beijing from the perspective of work–residence separation is provided, allowing assessment of the dynamic interaction between transportation infrastructure supply and urban growth.

The remainder of this paper is organized as follows. In Section 2, the proposed method is formulated. In Section 3, the materials and methods for this study are described, a case study presented, and results and findings analyzed and discussed. Conclusions and future work are discussed in Section 4.

## 2. Materials and Methods

In this section, the AFC system and mobile phone data are briefly introduced, and the fractal dimensions are presented in detail. In particular, the URT passenger flow assignment model was formulated to classify URT passengers, revealing thee residents’ travel demand by URT and pointing to the evolutionary rule of BBS.

### 2.1. Automatic Fare Collection (AFC) Systems

**Figure 1:**
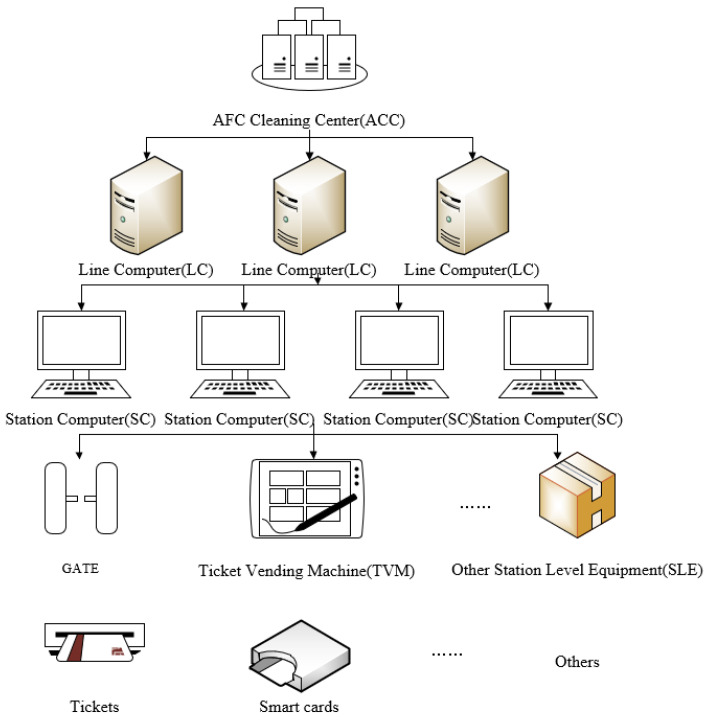
Five hierarchical levels of automatic fare collection (AFC) system.

The automatic fare collection (AFC) systems are an important part of URT, which can not only provide passengers with convenient, humanized services such as a quick and easy ticket checking process, but also providing operators with an automatic management platform containing ticket production, ticket sales, ticket inspection, financial, statistical analysis, audit and other functions. As displayed in Figure 1, a typical AFC system consists of five hierarchical levels: AFC cleaning center (ACC), line computer (LC), station computer (SC), station level equipment (SLE), and smart cards and tickets.

The smart ticket and card contain an integrated circuit (IC) clip (a type of microsensor) inside, so if a passenger touches a smart ticket or card to a turnstile when boarding or alighting, the sensor in the turnstile will record and respond some necessary information such as card code, boarding/alighting station, boarding/alighting time, and some other useful information. Then, this information will be transmitted to the SC, LC, and finally to the CC. Meanwhile, the AFC data do not contain the passenger’s private information such as passenger’s name, age, occupation, etc. Therefore, this does not invade people’s privacy.

The AFC system can not only be employed by operators to calculate the fares from passengers directly, but the massive data collected by AFC system can also help researchers to detect the urban mobility structure and travel demand of residents.

### 2.2. Mobile Phone Data

The mobile phone data applied in this paper contained two types of datasets. The first, which presents ODs between different geographic coordinate points at different times for a single day, contains detailed information about the individual passengers’ trips including time, latitude and longitude of origin, latitude and longitude of destination, and ODs. The second presents the numbers of people per geographic points at different times on a single day including accurate time, latitude and longitude of geographic coordinate points, and numbers of people.

### 2.3. Fractal Dimensions

#### 2.3.1. Volume Dimension

Along with economic and urban development, urban mobility demand fluctuates around a certain value in a period [30]. In a circular area of radius r, load level in a topological network and in a demand-weighted network can be defined, respectively, by

(1) $$S(r)=C_{S}*r^{D_{S}}$$

(2) $$V(r)=C_{V}*r^{D_{V}}$$

where S(r) and V(r) represent the number of stations and their total demand, respectively, within the area of radius r; $C_{S}$ and $C_{V}$ are constant coefficients; $D_{S}$ and $D_{V}$ are the station dimension and volume dimension using the fractural method, respectively.

The volume dimension $D_{V}$ depicts the supply level change from the city center to the surrounding areas. Using the derivative transform, the spatial attenuation formula for demand-weighted network volume is obtained by

(3) $$\rho (r)\propto r^{D_{V}-d}$$

where d is the Euclidean dimension d=2. If $D_{V}<2$, the density distribution of transportation supply decreases from the city center to the suburbs, with capacity decreasing gradually. If $D_{V}=2$, the distribution of capacity is homogeneous. If $D_{V}>2$, the network has a heterogeneous density distribution, which is common in multicenter cities.

For a detailed reflection of demand density distribution, inbound passenger flow and outbound passenger flow are used to define the inflow volume dimension $D_{V}^{in}$ and outflow volume dimension $D_{V}^{out}$, respectively,

(4) $$V^{in}(r)=C_{V}^{in}*r^{D_{V}^{in}}$$

(5) $$V^{out}(r)=C_{V}^{out}*r^{D_{V}^{out}}$$

where $V^{in}(r)$ and $V^{out}(r)$ are the cumulative flow volume in each circular area with radius r of inflow and outflow, respectively.

#### 2.3.2. Traffic Impedance Dimension

The sectional passenger flow and interstation distance can reflect the efficiency and accessibility of a transport network. The distance $d_{ij}$ between two stations i and j is denoted as the real distance between two stations. The traffic impedance dimension in terms of interstation distance $D_{I}^{d}$ is defined in Equation (6), and the impedance dimension represented by sectional flow $D_{I}^{f}$ is defined in Equation (7),

(6) $$I^{d}(r)=C_{I}^{d}*r^{D_{I}^{d}}$$

(7) $$I^{f}(r)=C_{I}^{f}*r^{D_{I}^{f}}$$

where $I^{d}(r)$ is the cumulative interstation distance in each circular area with radius r and $I^{f}(r)$ is the cumulative sectional passenger flow in each circular area of radius r.

Rail lines are normally bidirectional: upstream and downstream, both predefined. For detailed description of flow direction, upstream passenger flow and downstream passenger flow are used to define the upstream volume dimension and the downstream volume dimension, respectively,

(8) $$V^{up}(r)=C_{V}^{up}*r^{D_{V}^{up}}$$

(9) $$V^{down}(r)=C_{V}^{down}*r^{D_{V}^{down}}$$

where $V^{up}(r)$ and $V^{down}(r)$ is the cumulative flow volume in each circular area with radius r of upstream and downstream, respectively.

#### 2.3.3. Branch Dimension

Volume dimension $D_{V}$ represents the URT network’s capacity and the traffic impedance dimension’s accessibility. The connection relationship is fully represented by these two dimensions’ indicators, but because the development level of the network structure itself is not captured, the branch dimension is introduced to describe the complexity of the network structure. The subway lines can be divided into branches, with branch dimension $D_{B}$ defined as

(10) $$B(r)=C_{B}*r^{D_{B}}$$

where B(r) is the number of branches in the circular area described by radius r. The larger B(r), the more branches of lines and the better the URT network supply.

#### 2.3.4. Fractal Dimension Consistency Index

Passenger flow and the topological network interact in a URT system. To examine the interrelation of topological structure and passenger flow, the fractal dimension consistency index γ is chosen for quantitative analysis [31],

(11) $$\gamma =\left\{ \begin{array}{ll} 1-\frac{\left|D_{S}-D_{V}^{in}\right|}{D_{V}^{in}}, & D_{S}\leq 2D_{V}^{in}\\ 0, & D_{S}>2D_{V}^{in} \end{array}\right.$$

where $D_{S}$ is the station dimension and $D_{V}^{in}$ is the inflow volume dimension. The larger the value of γ, the greater the consistency between the network supply and the demand of passenger flow.

### 2.4. Subway Passenger Flow Assignment

To acquire reliable sectional passenger flow data, an effective dynamic traffic assignment method is proposed that takes into account the passengers’ heterogeneity.

#### 2.4.1. Passenger Clustering

The temporal and spatial characteristics of passengers are indicators of passenger heterogeneity and can be considered for passenger clustering. The temporal features are represented by the travel days within a period for as long as possible considering data accessibility—at least a month. Regular passengers can be selected by trip frequency. The spatial features are characterized by spatial consistency based on the diversity of the station visited. The proportion of the most frequent origins for each passenger can be calculated by

(12) $$PR=\frac{m_{i,\max}}{\sum_{i=1}^{n}m_{i}}$$

where $m_{i,\max}$ is the travel time of the most frequently visiting origin station; n is the total travel time of all origin stations for one passenger; and $m_{i}$ is the travel time for origin station $O_{i}$. We took passengers with PR>α(α>0) as regular travel passengers for the following study.

For the regular travel passengers, a topic model and *K*-means were adopted for further clustering. Like the irregular passengers, they were classified as one clustering, with application of the clustering method not needed. First, the topic model was used to calculate the probability distribution of travel regularity for each passenger. In this study, each passenger can be regarded as a document containing a large number of words, which reflect the characteristics of the passengers’ time of station entry such as Friday at 10 a.m. The number of trips of passengers at different times indicates the corresponding topic, representing the probability of passengers’ preferring to travel at different times, whether morning, midday, or evening. The formulas for the topic model are described as

(13) z~M(1,π)

(14) $$u|z\sim M\left(D,\beta_{z}\right)$$

(15) $$p\left(u\right)=\underset{z}{\sum }p\left(z\right)\overset{D}{\underset{d=1}{\prod }}p\left(u_{d}|z\right)$$

where *z* denotes the topic; π the proportion of topic distribution; M the polynomial distribution; D total travel frequency for one topic; and β weekly travel regularity distribution for one topic; Equation (15) expresses the Expectation-Maximum (EM) algorithm and allows calculation of π, β.

Based on the topic model, the probability distributions of each passenger for different topics can be calculated, reflecting the probability distributions that correspond to each passenger’s various travel time patterns. Then, the probability distribution of different travel patterns of each passenger is taken as its eigenvalue, with the *K*-means algorithm used to cluster passengers.

#### 2.4.2. Passenger Travel Generalized Cost Function

Passenger path choices are affected by various factors, so that full consideration of impact factors, and reasonable quantification of them, is essential to the path choice model. We chose traveling time cost, interchange time cost, URT network familiarity, and degree of congestion as the key factors. For one OD pair rs, for a passenger in class n, the set of possible paths is $K_{n}^{rs}$, with the total cost of path k expressed as

(16) $$\begin{array}{ll} D_{k,n}^{rs} & =C_{k,n}^{rs}+\varepsilon_{k,n}^{rs}\\ & =\gamma \sum_{l}{\bar{T}}_{rs,k}^{l}+\sum_{i}{\bar{E}}_{i,n}^{l,m}+\varepsilon_{k,n}^{rs}\\ & =\gamma \sum_{l}\sum T_{i,j}^{l}*\delta_{ij,k}^{rs}*\left(1+Y\left(x_{w}\right)\right)+\\ & \;\sum_{i}\alpha_{n}*{\left(e_{i,k}^{rs}\right)}^{\beta_{n}}*\left(w_{i,n}^{l,m}+0.5*f_{m}\right)*\varphi_{i,k}^{rs}*\eta_{l,k}^{rs}*\eta_{m,k}^{rs}+\varepsilon_{k,n}^{rs} \end{array}$$

where $C_{k,n}^{rs}$ is the quantitative cost, which consists of in-vehicle time $\sum_{l}{\bar{T}}_{rs,k}^{l}$ and interchange time $\sum_{i}{\bar{E}}_{i,n}^{l,m}$; $\varepsilon_{k,n}^{rs}$ is the stochastic cost error; and interchange time $\sum_{i}{\bar{E}}_{i,n}^{l,m}$ comprises walking time $w_{i,n}^{l,m}$ and waiting time $0.5*f_{m}$ ($f_{m}$ is the headway, and assuming most of the service are regular). Walking time is obtained through field research, which is affected by station type, differences in transfer mode, transfer distance, and transfer height, whereas waiting time is calculated by headway, as obtained from the Beijing subway map. $e_{i,k}^{rs}$ is the accumulative interchange time of OD pair rs for path k at station i; $\alpha_{n}$ and $\beta_{n}$ are the parameters needing estimation; $\delta_{ij,k}^{rs}$, $\varphi_{i,k}^{rs}$, $\eta_{l,k}^{rs}$, $\eta_{m,k}^{rs}$ represent the affiliation relationship between sections or interchange stations or lines and path k, with a value of 1 indicating that the foregoing segments belong to path k; and $Y\left(x_{w}\right)$ denotes the degree of congestion, producing the expression

(17) $$\begin{array}{c} Y\left(x_{w}\right)=\left\{ \begin{array}{cc} 0, & x_{w}\leq p_{n}\\ A{\left(\frac{x_{w}-p_{n}}{p_{n}}\right)}^{\eta }, & p_{n}<x_{w}<p_{c}\\ A{\left(\frac{p_{c}-p_{n}}{p_{n}}\right)}^{\eta }+B{\left(\frac{x_{w}-p_{c}}{p_{n}}\right)}^{\psi }, & x_{w}\geq p_{c} \end{array}\right. \end{array}$$

where $x_{w}$ is the actual load of each vehicle; $p_{n}$ is the number of seats; $p_{c}$ is the rated load factor of each vehicle, when $x_{w}$ is smaller than $p_{n}$, which means there are seats for all passengers and the influence of congestion degree is 0; when $x_{w}$ is larger than $p_{n}$ and smaller than $p_{c}$, it means that the degree of congestion is a little high; when $x_{w}$ is larger than $p_{c}$, it means that the degree of congestion is very high; A and η are the coefficients for average level congestion (second situation), B and ψ are the coefficients for serious congestion (third situation).

Perceived interchange time and path preference differ for different classes of passengers. Combined with the subway operating parameters C, the time-allowable deviation coefficient σ, the time coefficient in vehicle γ, the transfer time coefficient α, β and the joint distribution of prior probability can be denoted as π(C,σ,γ,α,β), the conditional probability as p(Γ|C,σ,γ,α,β) and the posterior probability distribution of each parameter as π(C,σ,γ,α,β|Γ). By comparing actual time set Γ with the theoretical time for each class of passenger, the parameters were calibrated step by step. The theory of Bayesian inference [18,19] is denoted as

(18) π(C,σ,γ,α,β|Γ)∝p(Γ|C,σ,γ,α,β)π(C,σ,γ,α,β)

Assuming that all parameters are independent of each other, the posterior distribution theoretical solution of each parameter could be represented as

(19) $$\begin{array}{l} \pi (C,\sigma ,\gamma ,\alpha ,\beta |\Gamma )\propto \\ \prod_{w\in W}\left(\prod_{\Gamma \in T_{w}}\left(\sum_{r\in R_{w}}h\left(\Gamma |r,C,\sigma ,\gamma ,\alpha ,\beta \right)f_{w}\left(r|C,\sigma ,\gamma ,\alpha ,\beta \right)\right)\right)\times \pi \left(C\right)\pi \left(\sigma \right)\pi \left(\gamma \right)\pi \left(\alpha \right)\pi \left(\beta \right) \end{array}$$

With regard to the arithmetic solution of the posterior distribution, the large volume of passenger flow data must be processed and several parameters calibrated. Accordingly, the algorithm MCMC [20] was chosen as a time-saving and computerized capacity feasible method. The MCMC algorithm comprises three stages: Metropolis–Hasting sampling, Markov chain analysis, and error analysis.

#### 2.4.3. Acquisition of Sectional Passenger Flow

Using the passenger clustering and parameter estimation method, passengers’ generalized travel cost function for each class of passengers was determined. Adopting MLM [32], the choice of each effective path was determined as

(20) $$p_{k,n}^{rs}=\frac{\exp\left(-\theta C_{k,n}^{rs}/C_{\min}\right)}{\sum_{p\in K_{rs}^{n}}\left(-\theta C_{p,n}^{rs}/C_{\min}\right)}\;\;\;\;\;k\in K_{rs}^{n},n\in N$$

where $C_{\min}$ denotes the least cost among all the effective paths’ costs and θ is the network familiarity level of the passengers.

Then, the passenger flow of each path $f_{k,n}^{rs}$ is estimated:

(21) $$f_{k,n}^{rs}=q_{n}^{rs}\cdot p_{k,n}^{rs}$$

Finally, the cumulative $f_{k,n}^{rs}$ is defined as the sectional passenger volume $x_{a}^{}$:

(22) $$x_{a}=\sum_{r}\sum_{s}\sum_{k}f_{k}^{rs}\cdot \delta_{a,k}^{rs}\;(1)$$

### 2.5. The Hybrid Model

**Figure 2:**
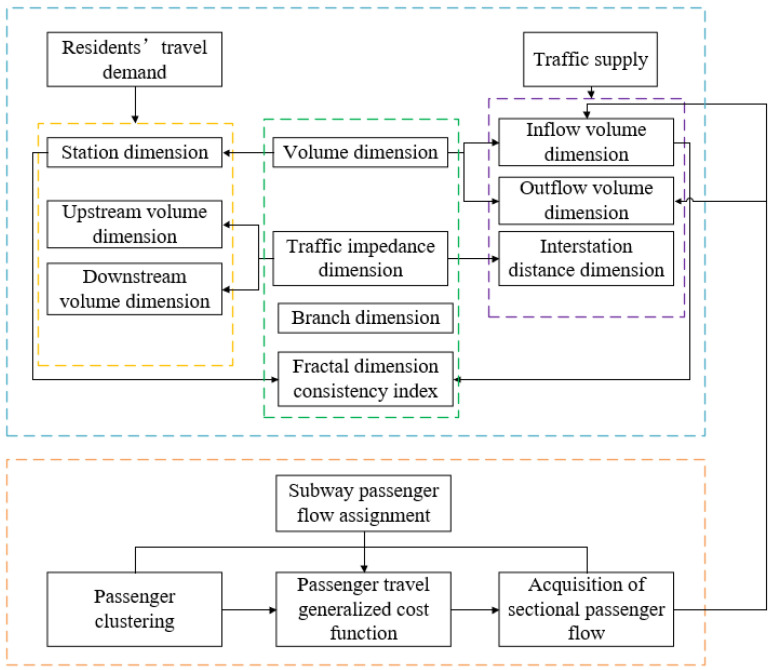
The flow chart of the combination of subway flow assignment and fractal dimensions.

The model combination of subway passenger flow assignment and fractal dimensions was proposed to extract the relationship and evolutionary rule connecting the residents’ travel demand and traffic supply. The flow chart of this hybrid model is displayed in Figure 2 and all variables are summarized in Table A1 (see Appendix A).

## 3. Empirical Study

### 3.1. Data Collection

In this study, we used BSS smart card data to extract the urban mobility demand of BSS. In addition, mobile phone location data were used to derive the proxy travel demand for the entire city. Each of the datasets is described as follows.

#### 3.1.1. BSS Smart Card Data

Six weeks of BSS smart card data recorded by automatic fee collection (AFC) systems were collected for each year during 2009 to 2018. The data included smart card ID, entry time, exit time, entry station, and exit station. Data were collected each day for the period from 5 a.m. to 11 p.m. The corresponding record numbers for each year are presented in Table 1.

The AFC data utilized in this paper only contains passengers who use smart card and does not contain other payments such as paper-ticket, NFC solutions, APP, etc. However, the percentage of passengers using smart cards is different in different lines and different stations, according to the data analysis results, which shows that the average percentage of passengers using smart cards was almost 70%, which means that the AFC data can reflect the general passenger flow of Beijing URT. Meanwhile, adopting AFC data may underestimate passenger demand due to fare evasion in some cities [33], however, there are security and gate systems for each station in Beijing URT systems, and it is well-staffed, so the number of fare evaders is low and cannot make a big difference to the research results.

#### 3.1.2. Mobile Phone Data

BBS demand does not give the whole picture of urban mobility in Beijing. It is difficult, if not impossible, to collect the data for all transport modes, but mobile phone data provides an alternative. Mobile phone data for 10 August 2016, were collected and used in this study to extract the entire travel demand in Beijing.

### 3.2. BSS Data Processing

The geographical subway lines and subway stations were acquired via the Baidu Map API. The BSS network was visualized in the GIS platform via embedded vector plotting. When processing AFC data, we found that some AFC data had errors such as missing some necessary data, however, the number of these incorrect data was very small and could not influence the research results. Therefore, we deleted these wrong data in the BSS data processing. Then, boarding and alighting demand for each station were retrieved directly from AFC data. Using the traffic assignment method, sectional passenger flow was also obtained.

**Figure 3:**
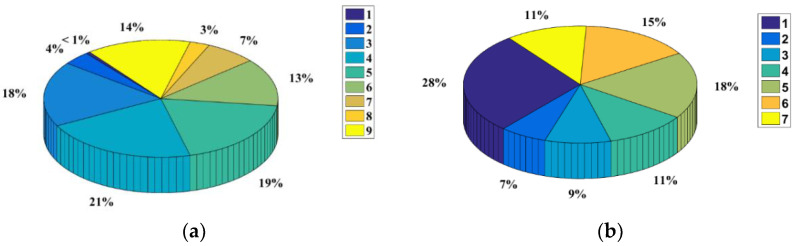
Percentages of passengers by topic model and shares of different classes: (**a**) percentages of passengers in different topics and (**b**) percentages of passenger clusters.

Taking the BSS travel data from 29 February to 3 April 2016 as an example, passengers who traveled more than six days in five weeks were regarded as regular travelers, who accounted for nearly 80% of total passengers on weekdays and 60% on weekends. For the spatial feature analysis, we took passengers with PR>0.3 to be regular passengers (accounting for 87.6%). Using the topic model, we calculated the probability distributions of each passenger for nine topics, obtaining the results shown in Figure 3a. According to Figure 3a, fewer than 1% of passengers covered only one topic, whereas the passengers covering four topics were the most numerous; passengers covering all nine topics accounted for 14%. The results show that almost all passenger travel was diverse and related to multiple topics. Passenger clustering results using the *K*-means algorithm are shown in Figure 3b, indicating that all passengers can be divided into seven clusters by their probability distribution of different travel time patterns. Parameter estimations are presented in Table 2. Using MLM, the demand for each section can be generated to allow for application of the fractal model.

**Figure 4:**
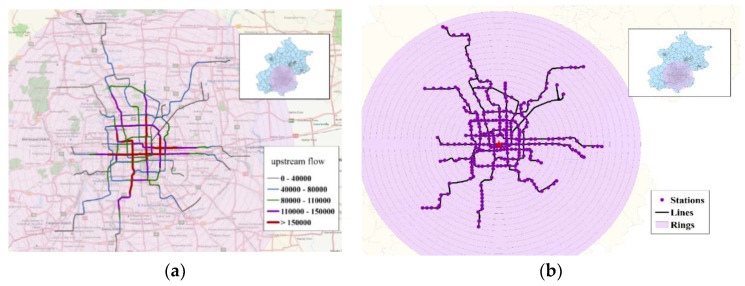
(**a**) Upstream flow in 2016 and (**b**) fractal units.

The geographical distribution of the sectional passenger flow is visualized in Figure 4a, with fractal dimensions calculated using GIS and Oracle. Considering the BSS as an undirected- and weighted-network, we applied the Dijkstra algorithm to calculate the network’s shortest path length between each pair of stations from 2016 to 2018. Among all stations, the lowest average shortest path length appeared at Tiananmen West Station and was chosen as the center of all rings, as shown in Figure 4b.

### 3.3. Fractal Analysis of BSS

#### 3.3.1. Station and Volume

**Figure 5:**
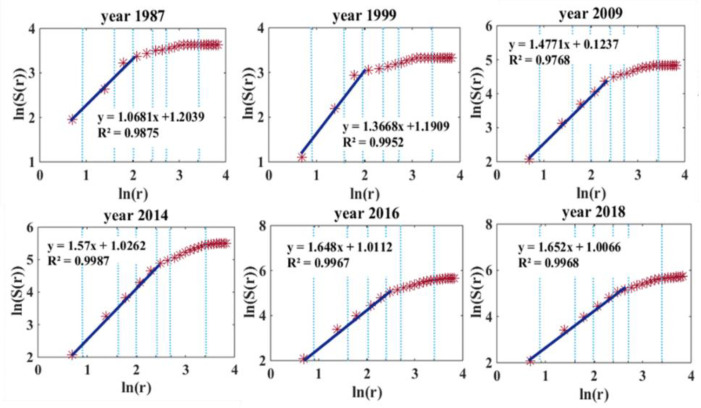
Fitting plots of station dimension for different years.

For the BSS, station dimensions, total volume, entry volume, and exit volume were respectively calculated using Equations (1)–(10). Figure 5 shows ln(*r*) and ln(*S*(*r*)) for different years, with the blue dotted lines denoting Beijing’s six ring roads.

This shows that the non-scale area is expanding from the inside of the third ring road to the fifth ring road (points scattered along a straight line), consistent with increases in station dimension $D_{S}$ (slope of the straight line) for the BSS network.

**Figure 6:**
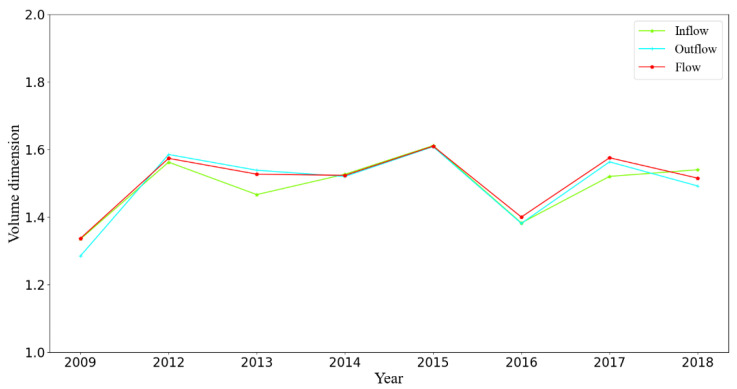
Volume dimension for each year.

Figure 6 summarizes the volume dimension $D_{V}$. All volume dimensions for each year were less than 2, indicating that demand density decreased from the city center to the suburb area, with demand commensurately lower in the suburbs than in the city center. This points out that suburb area could become a focus of government urban planning in the future such as tourist industry, breeding industry, etc.

The volume dimension $D_{V}$ in BSS was a bit lower than that of the London Underground and Paris Metro (around 1.7) [12], indicating that they are at different stages of development. We also observed a fluctuation in the indicators of the demand-weighted network from 2009 to 2018, and the non-scale area within the fourth ring underlines a gap between URT topology and rail transit demand.

#### 3.3.2. Traffic Impedance

**Figure 7:**
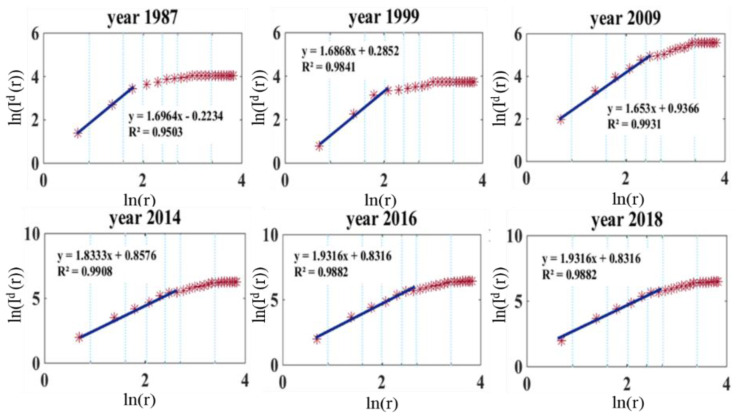
Interstation distance for select years.

Using the subway traffic assignment model and the OD matrix, we acquired the section load of each link between each adjacent station pair including the upstream and downstream flows. These sectional loads were incorporated in the dimension calculation. Figure 7 shows the interstation distance $D_{I}^{f}$.

An increasing trend of interstation distance without section load could be observed, indicating that the accessibility of the whole network increases with time. Note that this indicator was close to 2 within the fifth ring road, implying that the accessibility in the city center was well developed in its network structure. The interstation distance dimensions $D_{I}^{f}$ were all below 2 out of the fifth ring road, indicating that the density of the BSS network decreased from the center to the suburbs.

**Figure 8:**
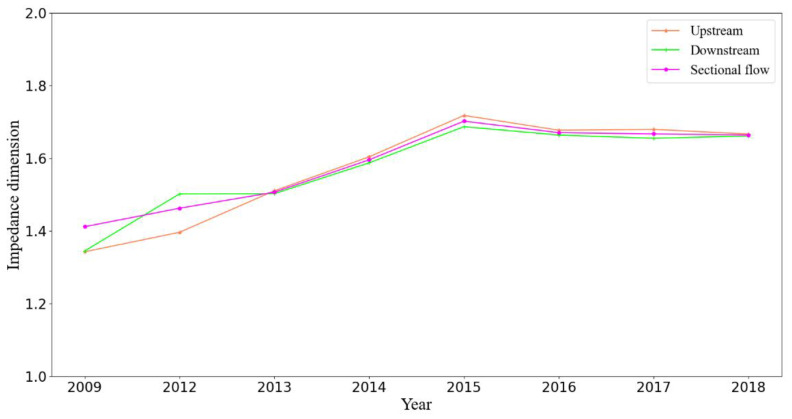
Impedance dimension with demand as a weight.

Taking into account the section load on the link produced the results shown in Figure 8, which demonstrated the same trend but a lower dimension than without demand. Such a result is common for a mono-centered city, in which the majority of demand is concentrated in the center area.

#### 3.3.3. Branch Dimension

**Figure 9:**
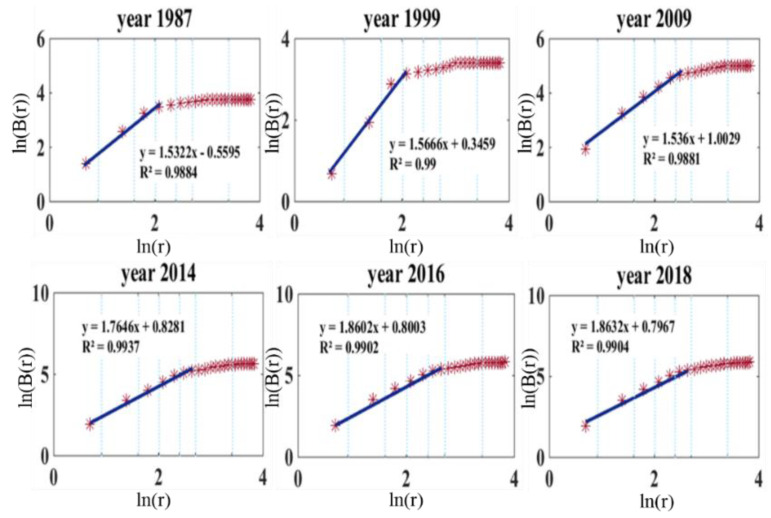
Fitting plots of branch dimension.

The branch dimension $D_{B}$ is used to evaluate the URT network’s development level. The higher the branch dimension, the more complicated the network and the greater the accessibility. Figure 9 shows the results for various years.

As revealed by ln(*B*(*r*)), increasing amounts of urban area are being covered by the BSS, and the fractal characteristic is becoming more and more obvious with time, having grown from 1.53 in 1987 to 1.86 in 2018.

#### 3.3.4. Fractal Dimension Consistency Index in BSS

**Figure 10:**
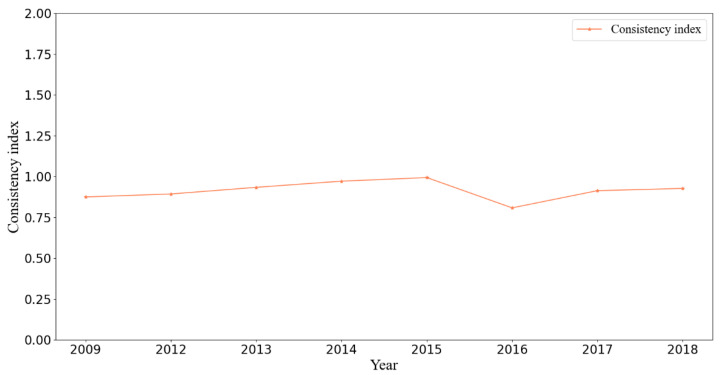
Fractal dimension consistency index.

Using Equation (11), Figure 10 shows the fractal dimension consistency index γ for each year. The consistency index γ was close to 1, indicating that the supply of transportation is strongly related to the demand of passenger flow. Furthermore, the index increased monotonically before 2015, peaking in 2015 before hitting its low point in 2016. The locations of newly built subway lines contributed to this pattern. Before 2015, construction of URT lines emphasized more than the city center. As the network matured in the city center, the focus began shifting toward remote suburban areas, with as many as three suburban lines opening at the end of 2015. The implications are twofold. First, these lines attracted very low demand, with a large train headway, especially at their opening. The low load level generates inconsistency between supply and demand. Second, these lines increase the suburban accessibility, attracting more residents to downtown areas, thereby increasing the spatial heterogeneity of demand. Thus, the consistency indicator decreased in 2016. As newly opened suburban lines cultivated demand, and thanks to the implementation of decentralizing policies in Beijing, residents even traveled to suburban areas for work. These inconsistencies were mitigated as time passed.

### 3.4. Analysis of Urban Travel Demand from Mobile Phone Data

Rail demand was used in the foregoing analysis, but is only part of the overall picture of urban mobility. To further investigate the role of rail in the residents’ daily transport, we must consider the whole picture. As collecting demand for all modes is quite difficult, we used demand extracted from mobile phone data as a proxy. Taking 2016 as an example, we estimated the population distribution of the whole city and derived travel demand using mobile phone data.

**Figure 11:**
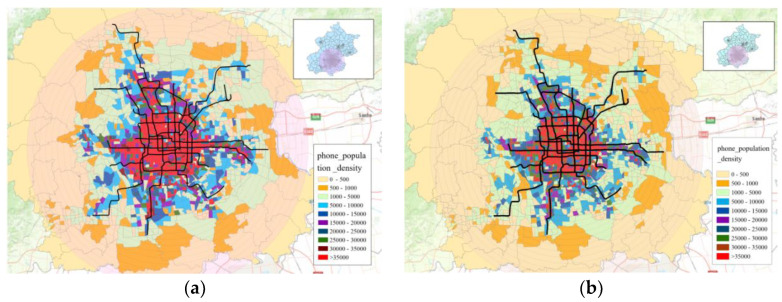
Population distribution (**a**) from 7 a.m. to 8 a.m. and (**b**) from 5 p.m. to 6 p.m.

As shown in Figure 11a, the urban population is distributed mainly outside the fourth ring road at the start of morning peak, scattered along the subway lines. In contrast, at the start of the evening peak, as shown in Figure 11b, the population is concentrated inside the fourth ring road and distributed along the subway lines. Such observations indirectly demonstrate that the BSS plays a major role in work–residence separation and has a strong gathering and guiding effect on passenger flow.

**Figure 12:**
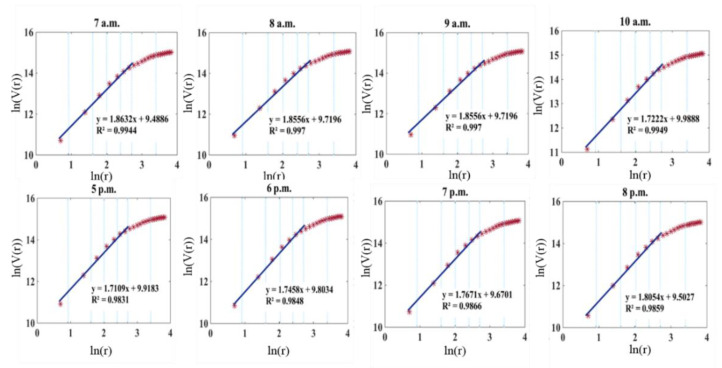
Volume dimension of population distribution deprived from mobile phone data.

Based on mobile phone data, Figure 12 shows ln(*V*(*r*)) for two peak periods in the day, the morning (7–10 a.m.) and evening (5–8 p.m.) peaks. The volume dimension $D_{V}$ from 8 a.m. to 9 a.m. was almost the same as from 9 a.m. to 10 a.m. The change rate of the volume in the morning peak was faster than that of the evening peak, demonstrating that the commuting period in the morning peak was more concentrated than that in the evening peak. For the evening peak, ln(*V*(*r*)) increased gradually, demonstrating that the residents returned to their dwellings after work within a longer time span.

**Figure 13:**
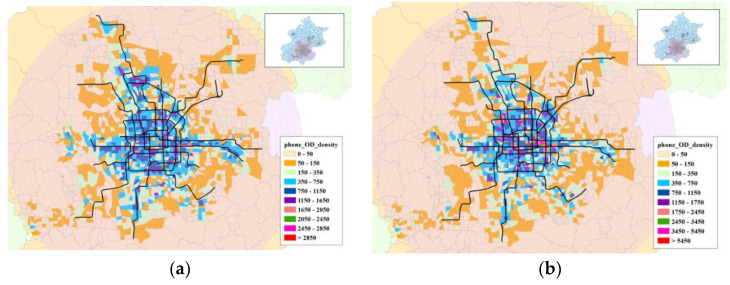
OD distribution in the morning peak, for (**a**) origin and (**b**) destination.

The ODs derived from the mobile phone data could help directly demonstrate spatial travel demand, revealing an evident trend of traveling from the outside city to the center of the city. Figure 13 shows the corresponding geographical OD distributions for the morning peak (7–10 a.m.).

This shows that the origins were concentrated mainly in the suburb areas, whereas the destinations were distributed mainly in the center of the city during the morning peak.

**Figure 14:**
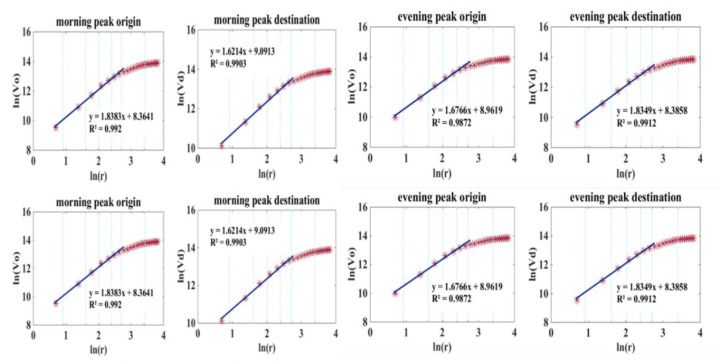
Volume dimension of mobile phone ODs.

According to Figure 14, for the morning peak origin and evening peak destination, the volume was similar to that of the population distribution. It can be inferred that the morning mobility origins of most urban residents are in the home, regardless of their occupation. Much as for the morning–evening comparison results of population distribution, it can be concluded that the imbalance between employment and housing is dramatic in Beijing.

We extracted urban resident distribution and traffic demand from mobile phone data. Different transport modes play different roles in transportation supply, and their volume dimensions should be compared from the perspective of fractal analysis.

### 3.5. Comparing BSS with Travel Demand Based on Mobile Phone Data

**Figure 15:**
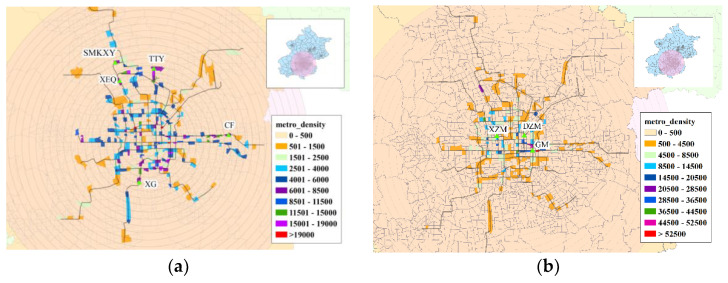
The density distribution of metro passengers (morning peak): (**a**) origin and (**b**) destination.

Figure 15 shows an obvious commuting pattern for subway passengers. Some typical residential areas are evident in Figure 15a such as TianTongYuan, ShengMingKeXueYuan, and XiErQi, and some work locations are identifiable in Figure 15b such as GuoMao, DaZhiMen, and XiZhiMen.

**Figure 16:**
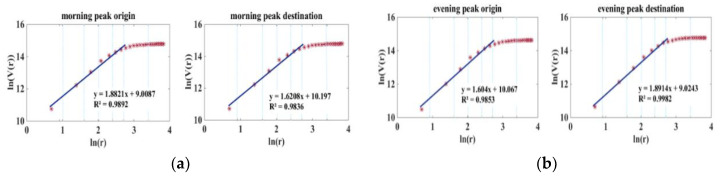
Volume dimension of BSS demand.

As Figure 16 shows, the volume dimension $D_{V}$ for morning peak origin and evening peak destination in BSS was larger than that of the population and the total OD pairs extracted from phone data. Furthermore, the volume dimension of the morning peak destination and the evening peak origin was lower than that of the population and total OD pairs. It can be concluded that subway-related mobility demand is more unbalanced than the average level of all modes, as reflected in the phone data. Due to the rail service’s speed, residents can choose to live far from their workplace, so that the subway system actually increases work–home imbalance.

## 4. Conclusions and Discussion

In this study, we proposed a fractal model with which to extract the relationship and evolutionary rule connecting resident travel demand and traffic supply using multisource data. We first presented a fractal model for addressing both the topologic network and the transportation demand on it. These methods can be used to reveal the level of development, volume capacity, accessibility, and consistency of the morphology network and the demand network. To acquire the sectional demand that is the input for fractal analysis, we further proposed a stochastic traffic assignment model based on passenger clustering. Using public transportation network data for Beijing, China, we presented a detailed analysis based on the proposed model. Furthermore, to investigate the role of BSS on overall urban mobility in Beijing, we extracted the population distribution and OD data from mobile phone data. We compared the fractal characteristics of a rail network with demand as a weight and the fractal features of proxy urban mobility based on phone data. Our results show that the transit network reveals more information than the topological network alone, especially in the context of network development. Comparison of the results of BSS and of using phone data as a proxy illustrates the role of rail transit in urban mobility. The main conclusions of this study thus include the following:

- Passenger flow is considered for comprehensive investigation of URT effectiveness. Demand-weighted network analysis reveals more information about the evolution of URT and its correspondence to urban mobility than a purely topological network.
- Non-scale area and self-similarity of the URT network in both the topological and the demand aspects is seen in Beijing’s public transportation systems, along with a nonlinear area in the log coordinates—namely, fractal degradation in the early years of URT growth. In addition, for the topological network, nearly all of the fractal dimensions is increasing. However, when demand is taken into account, irregular fluctuations can be seen for some fractal dimensions, revealing inconsistencies in URT demand and supply that offer network work planning insights unable to be captured by pure physical network analysis.
- Using phone-based population distribution data as a benchmark, the travel pattern of rail transit and its implications for urban mobility can be investigated and compared through fractal analysis. Different transit modes have markedly different roles in catering to commuting demand. URT normally plays a leading role because of its large capacity and exclusive right of way, and it actually increases job–work separation.

Some other aspects could be further investigated in future studies including the following: (1) The volume dimensions mentioned in this paper cannot depict the different development levels of a network in various directions within one fractal unit. The box-count dimension [34] could provide complementary descriptions for directional analysis of URT and urban expansion. (2) The fractal dimension calculated at the city scale of each transport mode is not accurate enough to provide detailed information or allow further comparison. In this paper, we used the passenger flow density distribution of each transportation analysis zone (TAZ) as supplementary information to offer additional detail, but the information provided by the density distribution is limited to use for comparison with the fractal dimensions. Thus, calculation of fractal dimensions at the TAZ level could be conducted in future studies.

## Figures and Tables

**Table 1:** BSS data description.

Year	Date	Records
2009	14 December~20 December	33,589,115
2013	4 March~10 March	34,224,238
2015	21 December~27 December	33,458,698
2016	19 December~25 December	33,581,774
2017	18 December~24 December	32,478,975
2018	18 September~24 September	32,558,423

**Table 2:** Parameter results.

Passenger Class	γ	$\alpha_{n}$	$\beta_{n}$	θ
Class 1	0.90	1.53	3.97	4.45
Class 2	1.33	3.68	3.01	3.87
Class 3	1.16	0.79	1.72	3.51
Class 4	1.19	0.84	1.64	3.12
Class 5	1.21	0.42	1.85	6.85
Class 6	1.11	3.98	3.77	2.16
Class 7	1.15	1.87	2.66	1.88

## Data Availability

Data sharing is not applicable to this article.

## Appendix A

**Table A1:** Summary of all variables.

Variables	Meanings
S(r)	Number of stations
V(r)	Cumulative flow volume
$D_{S}$	Station dimension
$D_{V}$	Volume dimension
$D_{V}^{in}$	Inflow volume dimension
$D_{V}^{out}$	Outflow volume dimension
$V^{in}(r)$	Cumulative inflow volume in each circular area with radius r
$V^{out}(r)$	Cumulative outflow volume in each circular area with radius r
$d_{ij}$	Distance between two stations i and j
$D_{I}^{d}$	Traffic impedance dimension in terms of interstation distance
$D_{I}^{f}$	Impedance dimension represented by sectional flow
$I^{d}(r)$	Cumulative interstation distance in each circular area with radius *r*
$I^{f}(r)$	Cumulative sectional passenger flow in each circular area of radius *r*
$V^{up}(r)$	Cumulative flow volume in each circular area with radius *r* of upstream
$V^{down}(r)$	Cumulative flow volume in each circular area with radius *r* of downstream
$D_{V}^{up}$	Upstream volume dimension
$D_{V}^{down}$	Downstream volume dimension
$D_{B}$	Branch dimension
B(r)	Number of branches in the circular area described by radius *r*
γ	Fractal dimension consistency index
$C_{k,n}^{rs}$	Quantitative cost
$\sum_{l}{\bar{T}}_{rs,k}^{l}$	In-vehicle time
$\sum_{i}{\bar{E}}_{i,n}^{l,m}$	Interchange time
$\varepsilon_{k,n}^{rs}$	Stochastic cost error
$w_{i,n}^{l,m}$	Walking time
$f_{m}$	Headway
$e_{i,k}^{rs}$	Accumulative interchange time of OD pair rs for path k at station i
$Y\left(x_{w}\right)$	Degree of congestion
$x_{w}$	Actual load of each vehicle
$p_{n}$	Number of seats
$p_{c}$	Rated load factor of each vehicle
Γ	Actual time set

## References

[1] Talib N.H., Hasnan K.B., Nawawi A.B., Abdullah H.B., Elewe A.M. (2020). Monitoring large-scale rail transit systems based on an analytic hierarchy process/gradient-based cuckoo search algorithm (GBCS) scheme. Urban Rail Transit.

[2] Obsie A., Woldeamanuel M., Woldetensae B. (2020). Service Quality of Addis Ababa Light Rail Transit: Passengers’ Views and Perspectives. Urban Rail Transit.

[3] Abutaleb A., McDougall K., Basson M., Hassan R., Mahmood M.N. (2020). The Impact of Transit-Oriented Shopping Mall Developments (TOSMDs) on Metro Station Ridership: Dubai Metro Redline. Urban Rail Transit.

[4] Screen D., Parkinson J., Shilton C., Rjabovs A., Marinov M. (2020). Data Analysis to Study Sub-threshold Delays Incurred by Tyne and Wear Metro Trains. Urban Rail Transit.

[5] Weerawat W., Samitiwantikul L., Torpanya R. (2020). Operational Challenges of the Bangkok Airport Rail Link. Urban Rail Transit.

[6] Sönmez H.Y., Öztürk Z. (2020). Effects of traffic loads and track parameters on rail wear: A case study for Yenikapi–Ataturk Airport Light Rail Transit Line. Urban Rail Transit.

[7] Potti P., Marinov M. (2020). Evaluation of Actual Timetables and Utilization Levels of West Midlands Metro Using Event-Based Simulations. Urban Rail Transit.

[8] Guerrieri M. (2020). Correction to: Tramways in Urban Areas: An Overview on Safety at Road Intersections. Urban Rail Transit.

[9] Christoforou Z., Chandakas E., Kaparias I. (2020). Investigating the Impact of Dwell Time on the Reliability of Urban Light Rail Operations. Urban Rail Transit.

[10] Mandelbrot B.B. (1983). The Fractal Geometry of Nature.

[11] Pavón-Domínguez P., Rincón-Casado A., Ruiz P., Camacho-Magriñán P. (2018). Multifractal approach for comparing road transport network geometry: The case of Spain. Phys. A Stat. Mech. Appl..

[12] Benguigui L. (1992). The fractal dimension of some railway networks. J. Phys. I.

[13] Batty M., Longley P.A. (1994). Fractal Cities: A Geometry of Form and Function.

[14] Batty M. (2008). The Size, Scale, and Shape of Cities. Science.

[15] Wang H., Luo S., Luo T. (2017). Fractal characteristics of urban surface transit and road networks: Case study of Strasbourg, France. Adv. Mech. Eng..

[16] Valério D., Lopes A.M., Machado J.A.T. (2016). Entropy Analysis of a Railway Network’s Complexity. Entropy.

[17] (2020). 2019 Beijing Transport Annual Report [EB/OL]. http://www.bjtrc.org.cn/List/index/cid/7.html.

[18] Chen Y. (2017). Multi-scaling allometric analysis for urban and regional development. Phys. A Stat. Mech. Appl..

[19] Lu Z., Zhang H., Southworth F., Crittenden J. (2016). Fractal dimensions of metropolitan area road networks and the impacts on the urban built environment. Ecol. Indic..

[20] Liu J.-L., Yu Z.-G., Anh V. (2015). Determination of multifractal dimensions of complex networks by means of the sandbox algorithm. Chaos: Interdiscip. J. Nonlinear Sci..

[21] Jalan S., Yadav A., Sarkar C., Boccaletti S. (2017). Unveiling the multi-fractal structure of complex networks. Chaos Solitons Fractals.

[22] Chriqui C., Robillard P. (1975). Common Bus Lines. Transp. Sci..

[23] Hassan M.N., Rashidi T.H., Nassir N. (2019). Consideration of different travel strategies and choice set sizes in transit path choice modelling. Transportation.

[24] Kim K.M., Hong S.-P., Ko S.-J., Kim D. (2015). Does crowding affect the path choice of metro passengers?. Transp. Res. Part A Policy Pract..

[25] Zhu W., Fan W.-L., Wahaballa A.M., Wei J. (2020). Calibrating travel time thresholds with cluster analysis and AFC data for passenger reasonable route generation on an urban rail transit network. Transportation.

[26] Zhu W., Zhou F., Huang J., Xu R. (2015). Validating rail traffic assignment models with cluster analysis and automatic fare collection data. Transp. Res. Rec..

[27] Rahbar M., Hickman M., Mesbah M., Tavassoli A. (2018). Calibrating a Bayesian traffic assignment model using smart card data. IEEE Trans. Intell. Transp. Syst..

[28] Rahbar M., Hickman M., Mesbah M., Tavassoli A. (2018). Determining effective sample size to calibrate a traffic assignment model: A Bayesian perspective. Transp. Res. Rec..

[29] Xu X., Xie L., Li H., Qin L. (2018). Learning the route choice behavior of subway passengers from AFC data. Expert Syst. Appl..

[30] Beijing Subway Subway Query 8 January 2019. http://www.bjsubway.com/en/.

[31] Wang Y.P., Chen K.M., Ma C.Q. (2008). Quantitative analysis of coordination between rail transit network configuration and urban form. J. Railw. Eng. Soc..

[32] Chen Z., Kuo L. (2001). A Note on the Estimation of the Multinomial Logit Model With Random Effects. Am. Stat..

[33] Barabino B., Lai C., Olivo A. (2020). Fare evasion in public transport systems: A review of the literature. Public Transp..

[34] Frankhauser P. (1990). Aspects fractals des structures urbaines. L’Espace Géographique.

